# Toxic shock syndrome complicated with symmetrical peripheral gangrene after liposuction and fat transfer: a case report and literature review

**DOI:** 10.1186/s12879-021-06777-2

**Published:** 2021-11-06

**Authors:** Zhiwan Liu, Wenjun Zhang, Boyu Zhang, Linhao Ma, Feng Zhou, Zheyuan Hu, Xiang Jie, Hong Gao, Xiaohai Zhu

**Affiliations:** 1 Department of Plastic Surgery, Shanghai Changzheng Hospital, No.415 Fengyang Road, Huangpu District, Shanghai, 200003 China; 2 Department of Emergency, Shanghai Changzheng Hospital, Shanghai, China; 3 Department of Plastic Surgery, Shanghai Tongji Hospital, Shanghai, China

**Keywords:** Liposuction, Toxic shock syndrome, Multiple organ dysfunction syndrome, Necrotizing soft tissue infection, Symmetrical peripheral gangrene

## Abstract

**Background:**

Liposuction is one of the most commonly performed aesthetic procedures. Toxic shock syndrome(TSS) is a rare, life-threatening complication. The incidence rate of TSS is very low in the plastic surgery field, especially after liposuction and fat transfer.

**Case presentation:**

A 23-year-old female patient was transferred to our emergency department from an aesthetic clinic with sepsis shock features after received liposuction and fat transfer. The patient underwent TSS, disseminated intravascular coagulation(DIC), multiple organ dysfunction syndrome (MODS), symmetrical peripheral gangrene (SPG), and necrotizing soft tissue infection of the buttocks in the next 10 days. Authors used a series of debridement and reconstructive surgery including vacuum sealing drainage (VSD) treatment, artificial dermis grafts,split-thickness skin grafts, amputation surgeries when her vital signs were stable. The patient experienced desquamation of the hand on the 26th day. The skin grafts survived and the function of both fingers and toes recovered. She was discharged 2 months after admission and was in good health.

**Conclusion:**

TSS is extremely rare in the field of liposuction and autologous fat transfer. The mortality rate of TSS is very high. Early diagnosis and operative treatment, as well as correction of systemic abnormalities, are the important keys to save a patient's life.

**Supplementary Information:**

The online version contains supplementary material available at 10.1186/s12879-021-06777-2.

## Background

With more and more people pursuing the beauty of body shape, liposuction and autologous fat transplantation have become one of the most commonly used cosmetic surgery in the world [1]. The common complications of liposuction include seroma, hematoma, infection, lymphoedema, hyperpigmentation, asymmetry [2, 3]. Lethal complications include necrotizing fasciitis, TSS, fat embolism syndrome, and even death [4–6]. However, most plastic surgeons and patients do not know much about the possible complications or comorbidities that may accompany such a procedure.

TSS was first described by Todd and co-workers in 1978 [7]. It can rapidly progress to shock and multiorgan failure. TSS is associated with postoperative wound infections that can occur in postoperative patients, in the previous reports the incidence of postoperative wound infection was very low if following clean elective procedures [8, 9]. 40–60% TSS is caused by Staphylococcus aureus, which presenting symptoms include high fever, diarrhea, nausea, a diffuse macular rash, and desquamation. Besides, most cases are reported to involve cosmetic plastic surgery such as chemical peeling, breast augmentation, rhinoplasty with and without nasal packing, abdominoplasty, and liposuction. SPG is defined as two or more limb ischemia injuries, no large vessel obstruction, or vasculitis. SPG is a rare but severe complication of DIC and is frequently associated with sepsis, which is 85% of the SPG caused by DIC [10]. SPG results in high rates of amputation and mortality; in particular, amputation is a serious problem for survivors [11, 12].

The incidence rate of TSS with SPG after liposuction and autologous fat transfer is extremely low, and there is no case was been reported worldwide, including in China. Here, we presented a young girl with TSS who experienced MODS, DIC, and SPG after liposuction and autologous fat transfer.

## Case presentation

### Case

A 23-year-old girl underwent liposuction of the thighs and extensive autologous fat transfer of buttocks, breasts, and face on 7 October 2020. 6 h after the operation, she had a high fever, hypotension, chills, vomiting, and bilateral thigh bleeding. The patient was thereafter admitted to our emergency room at night. On physical examination, her body temperature was 39.7℃, heart rate was 135 beats/minute, and blood pressure was 83/39 mmHg. Several small scattered incisions from the liposuction and lipotransfer process were observed in the thighs, buttocks, breasts, and face. Besides, petechial skin lesions on the region of the back, lumbosacral, buttocks, posterior thighs with swelling and tenderness, and diffusely distributed erythematous lesions appeared at her toes and hands.

The patient’s laboratory data were notable for a white cell count (WBC) of 2.8 × 10^9^ cells/L(normal:3.5–9.5 × 10^9^/L), hemoglobin 49 g/L(normal:115–150 g/L), and decreased platelets at 49 × 10^9 ^cells/L(normal:125–350 × 10^9^/L,). Blood coagulation studies revealed prothrombin time (PT) of 34.2 s(normal:9.8–12.1 s), activated partial thromboplastin time (APTT) of 131.6 s(normal:22.7–31.8 s), international normalized ratio (INR) 3.38(normal:0.79–1.14), D-dimers 70.40ug/ml(normal: < 0.55ug/mL), fibrinogen level was undetectable. Her potassium was 2.86 mmol/L(normal: 3.5–5.5 mmol/L), calcium was 1.57 mmol/L(normal: 2.25–2.75 mmol/L) and serum creatinine was 101umol/L(normal:46–92 umol/L). Liver function tests demonstrated albumin level was 13.5 g/L(normal:63.0–82.0 g/L); the total bilirubin level was 87.2 μmol/L(normal:2.0–22.0 μmol/L); the serum lactate level was 8.1 mmol/L (normal:0.5–1.6 mmol/L); the creatine kinase (CK) level was 918U/L(normal:30-135U/L), myocardial-bound creatine kinase (CK-MB) was 17U/L(normal:0-16U/L), muscle-type creatine kinase (CK-MM) was 901U/L(normal:0-137U/L)). An arterial blood gas analysis showed a potential of hydrogen (PH) of 7.37, a partial pressure of oxygen (PO_2_) of 165 mmHg, and a Partial Pressure of Carbon Dioxide (PCO_2_) of 29 mmHg. Cultures of blood, stool, and urine obtained before antibiotic therapy were negative.

She was additionally treated with vancomycin and imipenem, blood transfusion, massive fluid resuscitation, and a series of supportive therapy. During the next day, the patient’s condition worsened, a chest x-ray film showed massive infiltrations in both lungs. She was immediately admitted to the intensive care unit (ICU) and treated with continuous pumps of norepinephrine and metaraminol, imipenem, and vancomycin as empiric anti-infective, blood transfusions. On the second day after admission, the patient's condition deteriorated, and each test indicator continued to higher or lower than the normal range; CT showed pneumonia, pleural effusion, and gastric retention, and blood gas analysis suggested severe metabolic acidosis. Gastrointestinal decompression tube drainage gives about 1500 ml brown liquid.

She was in a state of persistent hyperthermia that her body temperature peaked at 40.1℃; On the third night after admission, the patient developed rapid heart rate (144 beats/min), shortness of breath (25 beats/min), and confusion; blood gas analysis indicated PH 7.31, PCO_2_ 50 mmHg, PO_2_ 43 mmHg, lactate 3.5 mmol/L, blood oxygen saturation(SpO_2_) 73%. Additionally, the patient’s urine was dark brown, and a large amount of exudation was drained from both thighs. Endotracheal intubation was performed with ventilator-assisted ventilation. On the 7th day after admission, bedside hemodialysis was performed because the patient's urine color continued to become dark, CK-MB, myoglobin, and infection indicators (C-reaction protein [CRP], Procalcitonin [PCT]) continued to rise. During the first 3 weeks after admission, a total of 3600 mL red blood cells, 1200 mL fresh frozen plasma, 4units(U) of platelet concentrate, 42U of cryoprecipitate coagulation factor, and large amounts of human fibrinogen and prothrombin complexes were used. The patient has gradually improved after positive and effective treatment, and the test indicators have gradually returned to the normal range (Additional file 1, which demonstrates the changes of patient’s body temperature and lab test indicators from day 1 through day 14 after admission).

**Fig. 1:**
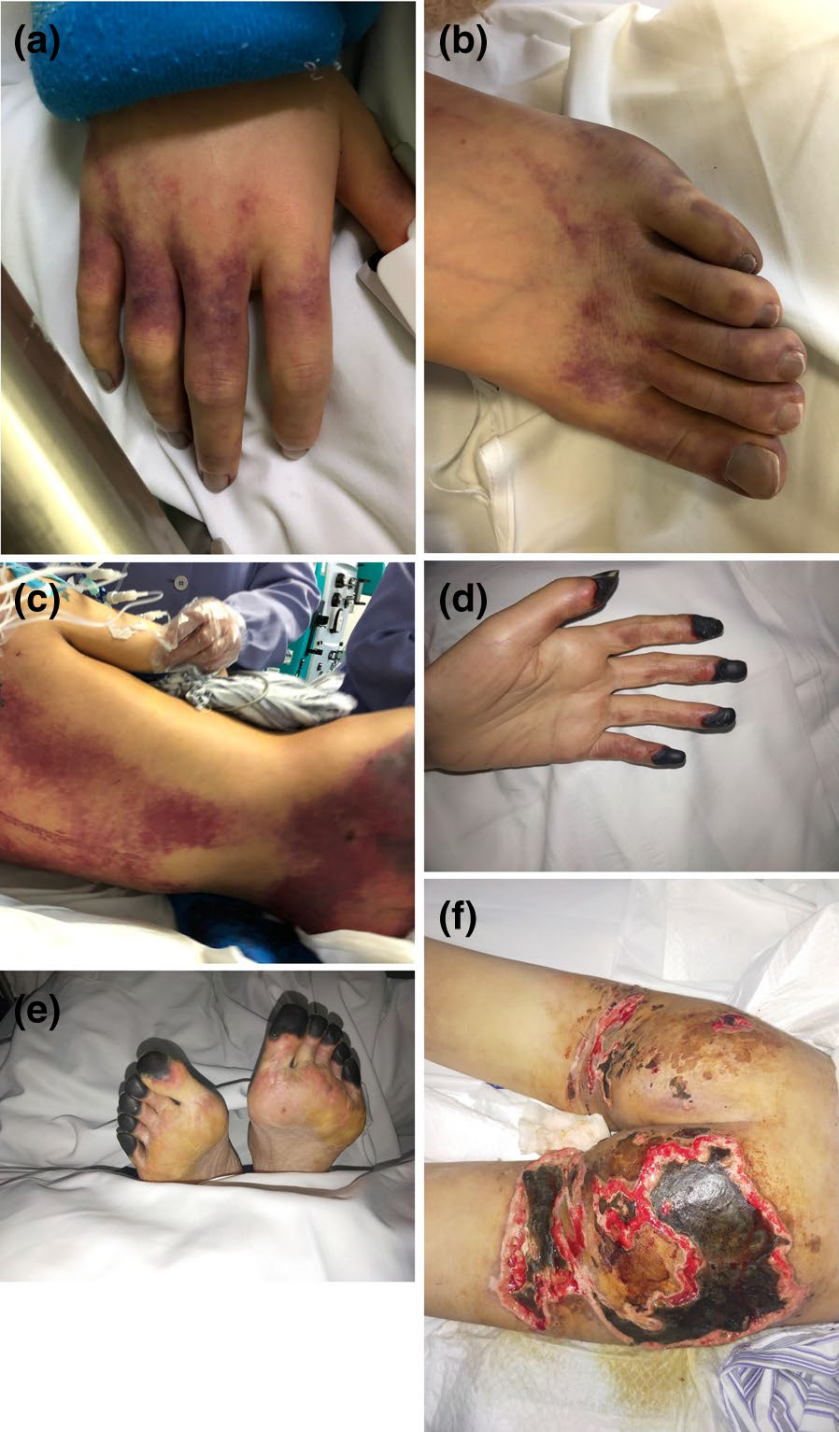
Petechial skin lesions appeared on her hands (**A**), toes (**B**), lumbosacral(**C**), buttocks (**C**), and posterior thighs (**C**) on the first day. Symmetrical peripheral gangrene of her fingers (**D**), toes (**E**), and necrotizing soft tissue infection on her buttocks (**F**) on hospital day 26

**Fig. 2:**
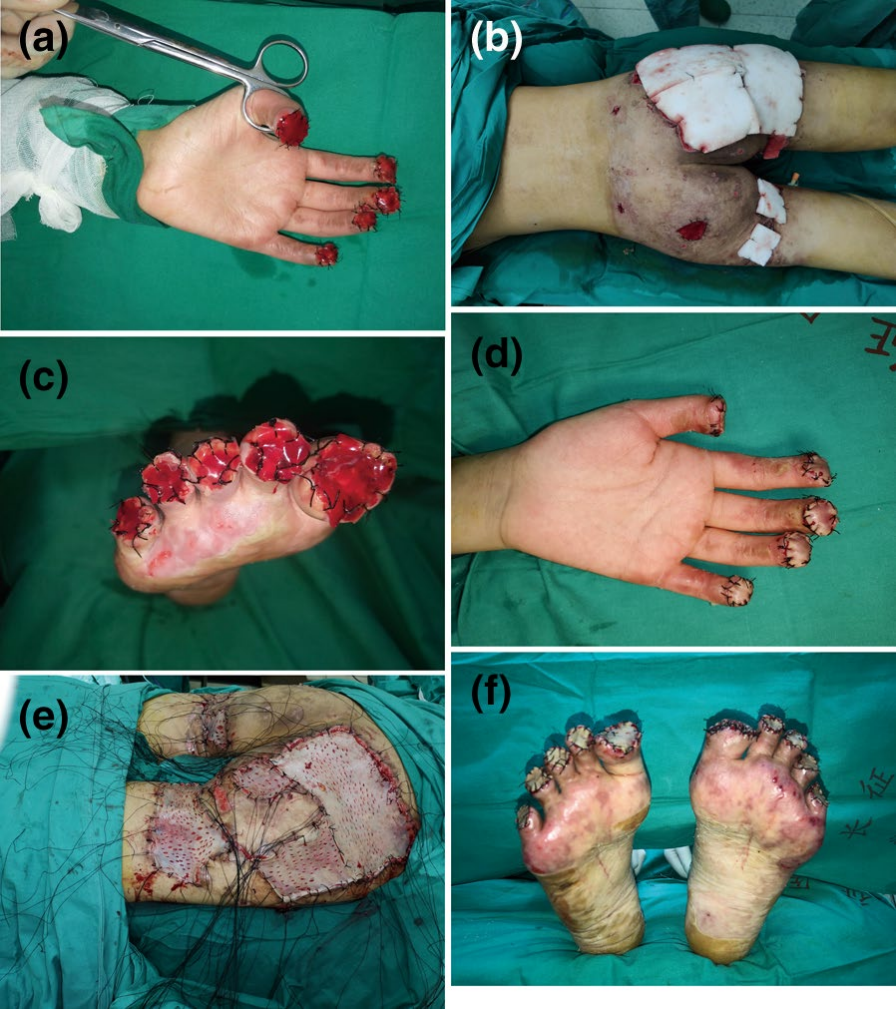
The reconstruction operation was completed in 4 stages. In the first stage, **A** surgical amputation of her fingers gangrenous areas on the peripheral extremities was performed and covered with artificial dermis grafts; **B** buttocks of the patient were debridement and treated with VSD. In the second stage, **C** toes amputation was performed and covered with artificial dermis grafts. In the third stage, the hands **D** and buttocks **E** of the patient were covered with split-thickness skin. At the last stage, **F** the toes of the patient were covered with split-thickness skin

Although the erythematous rush has subsided, peripheral ischemia appeared on her fingers and toes and gradually resulted in gangrene (Fig. 1). What’s worse, her buttocks occurred necrotizing soft tissue infection. By the time her body was strong enough to withstand surgery and the gangrenous areas became demarcated, surgical amputation of fingers and toes gangrenous areas on the peripheral extremities was performed; both fingers and toes were secondly covered with split-thickness skin after firstly artificial dermis grafts; her buttocks were grafted with split-thickness skin after twice full debridement and treated with VSD (Fig. 2). During the period, antibiotics (vancomycin, imipenem, teicoplanin, tigecycline, sulperazone, polymyxin, Linezolid, phosphonomycin) and antifungal drugs (Caspofungin, Fluconazole) were replaced according to the patient's sputum culture, wound secretion culture, and drug sensitivity.

**Fig. 3:**
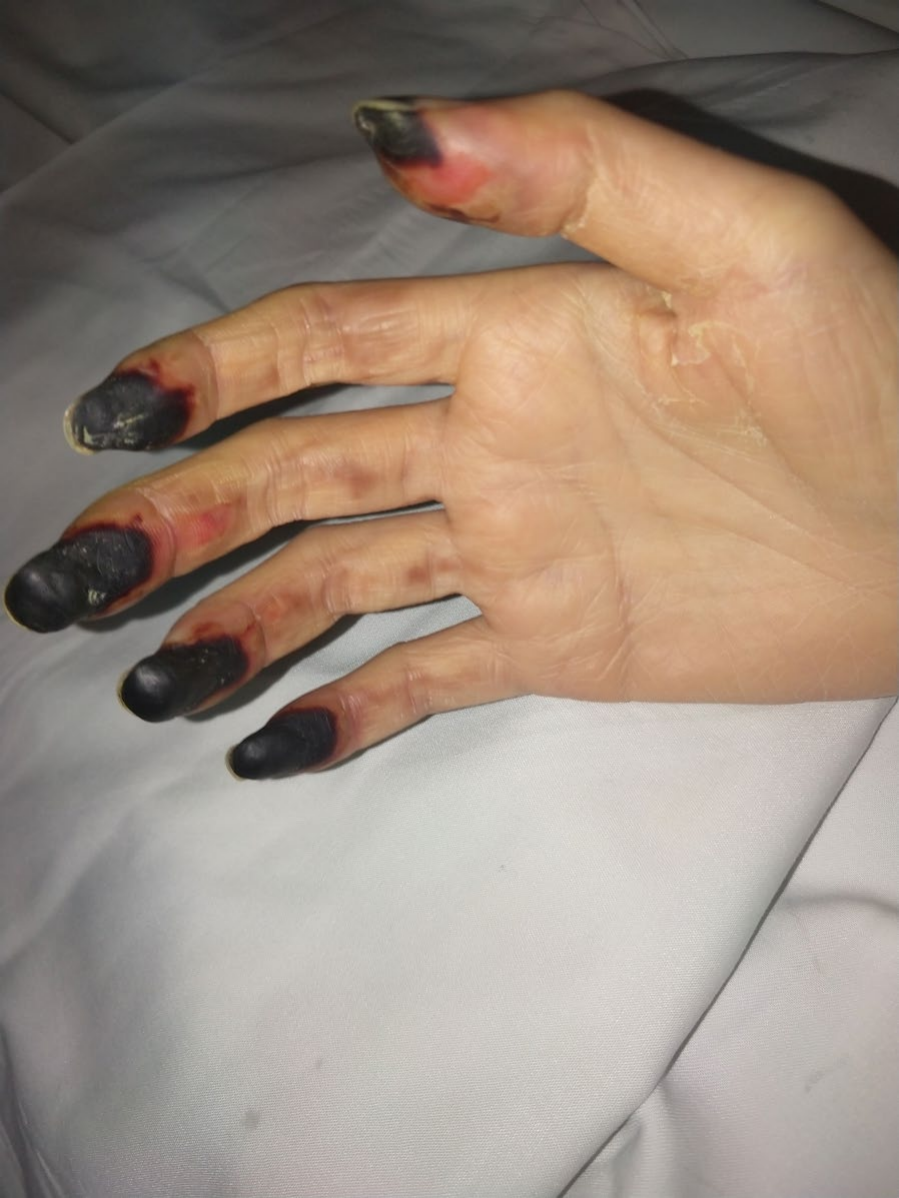
Desquamations of the patient’s hands were observed on hospital day 26

**Fig. 4:**
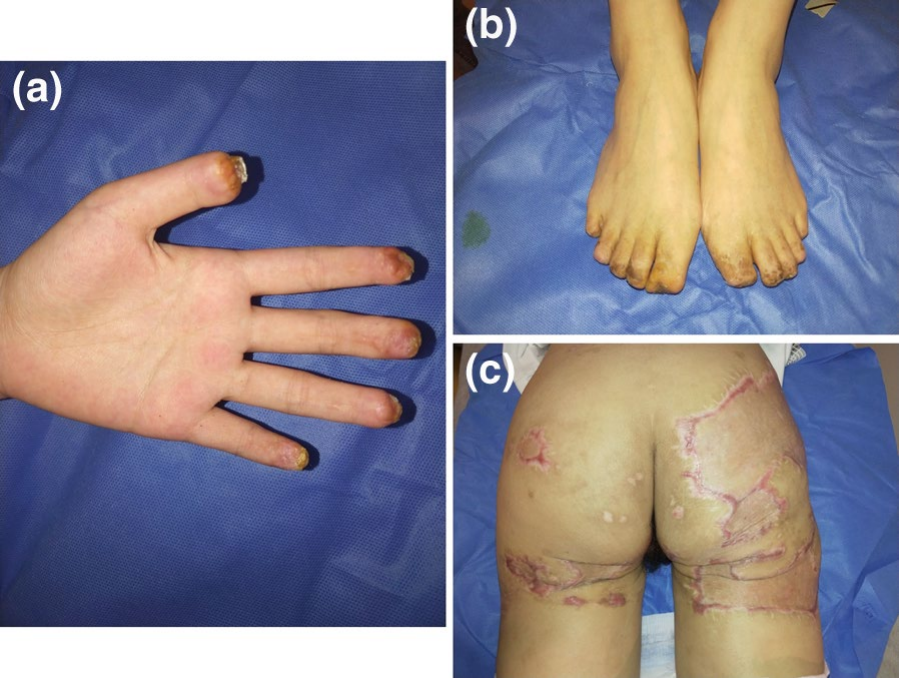
At the last follow-up after 4 months the hand (**A**), toes (**B**), and buttocks (**C**) of the patient were improved significantly

Blood cultures were persistently negative for the growth of organisms, but Candida tropicalis was identified by the untargeted next-generation sequencing of sputum; Klebsiella pneumonia was identified by the untargeted next-generation sequencing of blood; Acinetobacter baumannii, Proteus mirabilis, and Klebsiella pneumonia were detected in the buttock subcutaneous effusion culture. The patient experienced desquamation of the hand on the 26th day after admission (Fig. 3). The skin grafts survived and the function of both fingers and toes recovered well. She was discharged 2 months after admission and was in good health at the last follow-up (Fig. 4).

## Discussion and conclusions

Liposuction and autologous fat transfer are the most commonly performed cosmetic surgeries over worldwide. Most plastic surgeons and patients neglect its dangers, which the common clinic complications just include slight infection, bleeding, hematoma, seroma, and lymphoedema [2, 3]. However, that is not the case. As with any surgical procedure, liposuction still imparts potential morbidity and mortality. Major potentially life-threatening complications of liposuction include necrotizing fasciitis, TSS, pulmonary embolism, toxicity or drug interactions, and visceral organ perforation [4–6, 13]. TSS and necrosis fasciitis commonly are severe infections. Compared with the common postoperative complications after liposuction, TSS is very rare and relatively unknown to most plastic surgeons. During the past 30 years, only a few pieces of literature on TSS associated with liposuction have been reported [14–17]. TSS is a life-threatening infection characterized by a rapid progression of the disease. Since patients undergoing cosmetic surgery are basically healthy and demanding, any kind of fatal complication was hard to be accepted.

TSS is a multi-system, life-threatening disease, mainly caused by superantigen toxin-producing strains of Staphylococcus aureus and Streptococcus pyogenes [18–20]. Its clinical manifestation includes high fever, rash, vomiting, diarrhea, and multiple organ failure. The circumstances that cause TSS are more complex. Bacteria are the most common causative agents underlying postoperative TSS. Staphylococcus aureus, S epidermidis, Streptococci A and B, Streptococcus pyogenes, Klebsiella pneumonia, Bacillus are most often implicated. Corynebacterium, Pseudomonas aeruginosa, Escherichia coli, and Enterobacteriaceae are also occasionally implicated [9, 14, 21–29]. In the last few decades, TSS has been mainly associated with menstruating women who use intravaginal tampons [19, 30]. But with the rapid development of cosmetic surgeries, TSS has been observed a trend in increasing frequency in plastic surgical patients [31, 32].

The clinical diagnosis of TSS is based on a series of signs and symptoms, meanwhile, other diseases and causes that could lead to a septic shock-like state need to be excluded. The differences between TSS and common postoperative wound infections include a short incubation period, the rapid development of multiple system organ failures, and a series of dermatologic manifestations, requiring large amounts of fluid resuscitation and vasopressor to maintain blood pressure [8].

The constellation of our patient’s symptoms met with the TSS 2011 Case Definition by the Centers for Disease Control and Prevention. According to the standards supported by the Centers for Disease Control and Prevention in 2011 (Additional file 2, which provides detailed diagnostic criteria for TSS) [33], an obvious feature of postoperative TSS(Other Than Streptococcal) is that signs of local wound infection rarely occur. Blood, urine, and throat cultures are characteristically negative. Positive blood culture results are rare in staphylococcal TSS with less than 5% positive [1]. Despite a high fever and markedly increased white blood cell count, CRP, and PCT suggesting severe infection, the patient's blood culture results were consistently negative.

When we reviewed the previous literature, we found that this is might be the second reported case of SPG associated with TSS. The first case of SPG complicating TSS occurred in a young woman who is in lactation, she just experienced abortion surgery two months ago [34]. But our case might be the first case report of SPG associated with TSS in the field of plastic surgery. Most SPG relates to the use of vasopressors with DIC [35, 36]^.^ Hypotension due to sepsis is another suspected cause of SPG and is thought to be aggravated by the administration of hypotensive therapy [37]. There are no reports of SPG as sequelae of DIC in the setting of TSS. Intravascular thrombosis and infarction of the skin and distal extremities can be caused by DIC. The resulting low blood flow state can lead to thrombus occlusion of the microcirculation of the affected limb extremities [10]. The characteristics of SPG are symmetric necrosis of the skin and distal extremities, followed by gangrene in two or more distal sites without occlusion of the great arteries. About 18–40% mortality rate was reported, and survivors have a high frequency of multiple limb amputations [38]. Early amputation is contraindicated because secondary infection of necrotic tissue is uncommon and the boundaries of ischemic lesions occur over time [39]. No treatment has proved to be completely effective, so identifying and treating the underlying cause helps to arrest and prevent further progression in SPG [40]. Early recognition remains the key factor in SPG management.

In conclusion, early identification and timely treatment is the best way to reduce the mortality and sequelae of TSS. Controlling the source of infection through surgical debridement and abscess drainage is the first choice for initial and continuous treatment. When TSS is suspected, active fluid resuscitation, antibiotic treatment, and intensive care support should be taken immediately even if the patient has no signs of local infection. In addition, most of the patients who seek plastic surgery are healthy people who want to be better than before, but such serious complications are difficult for them and their families to accept. Therefore, it requires us to not only have a deep understanding of the occurrence and treatment of postoperative complications but also strictly abide by the principles of aseptic operation during the surgical operation.

## Supplementary Information

**Additional file 1: **Body temperature and laboratory parameters from day 1 to day 14 after admission.

**Additional file 2:** Centers for Disease Control and Prevention TSS (Other Than Streptococcal) Diagnostic Criteria.

## Data Availability

The datasets used and/or analysed during the current study are available from the corresponding author on reasonable request.

## Abbreviations

TSS: Toxic Shock Syndrome
DIC: Disseminated Intravascular Coagulation
MODS: Multiple Organ Dysfunction syndrome
SPG: Symmetrical Peripheral Gangrene
VSD: Vacuum Sealing Drainage
WBC: White cell count
PT: Prothrombin time
APTT: Activated partial thromboplastin time
INR: International normalized ratio
CK: Creatine Kinase
CK-MB: Myocardial-Bound Creatine Kinase
CK-MM: Muscle-type Creatine Kinase
PH: Potential of Hydrogen
PO_2_: Partial Pressure of Oxygen
PCO_2_: Partial Pressure of Carbon Dioxide
SpO2: Oxygen saturation
ICU: Intensive Care Unit
CRP: C-reaction protein
PCT: Procalcitonin
U: Units
