# Supplementary material for: Toxic shock syndrome complicated with symmetrical peripheral gangrene after liposuction and fat transfer: a case report and literature review
Source: BMC Infect Dis. 2021 Nov 6;21:1137. doi: 10.1186/s12879-021-06777-2 (PMC8571909; doi:10.1186/s12879-021-06777-2)
Supplement: Supplementary file 1 — Additional file 1: Body temperature and laboratory parameters from day 1 to day 14 after admission. [file 12879_2021_6777_MOESM1_ESM.docx]

**Additional file 1.** **Body temperature and laboratory parameters from day 1 to day 14 after admission.**

TABLE S1. Body temperature and laboratory parameters from day 1 to day 14 after admission

| **Parameter** | **Day 1** | **Day 2** | **Day 3** | **Day 4** | **Day 5** | **Day 6** | **Day 7** | **Day 8** | **Day 9** | **Day 10** | **Day 11** | **Day 12** | **Day 13** | **Day 14** |
| --- | --- | --- | --- | --- | --- | --- | --- | --- | --- | --- | --- | --- | --- | --- |
| Body temperature,℃ | 39.7 | 40.1 | 40 | 39.1 | 38.9 | 38.2 | 37.6 | 38.8 | 38.8 | 37.8 | 38.1 | 38.5 | 38.4 | 38.1 |
| Prothrombin time，S  （normal：9.8-12.1S） | 34.2 | 20.6 | 30.6 | 13.2 | 12.1 | 10.8 | 13.3 | 13.6 | 12.5 | 13.2 | 13.4 | 13.7 | - | 14 |
| INR  （normal：0.79-1.14） | 3.38 | 1.94 | 3.36 | 1.92 | 1.32 | 0.96 | 1.03 | 1.06 | 0.95 | 1.02 | 1.04 | 1.07 | - | 1.1 |
| APTT，S  （normal：22.7-31.8S） | 131.6 | 78.3 | 93.1 | 45.6 | 72.2 | 41 | 40.5 | 40 | 36.7 | 36.3 | 30.4 | 40 | - | 40.8 |
| Coagulation time，S  （normal：14-21S） | 52.2 | 21 | 48.4 | 25.5 | 75 | 27.1 | 20.5 | 21 | 21.6 | 24.2 | 19 | 16.9 | - | 16.2 |
| Fibrinogen, g/L  （normal：1.8-3.5g/L） | 0 | 1.79 | 0.78 | 1.79 | 1.99 | 3.12 | 2.74 | 2.75 | 2.49 | 2.27 | 1.89 | 2.07 | - | 2.3 |
| D – dimer, ug/ml  （normal：＜0.55ug/ml） | 70.4 | 53.78 | 100.9 | 40.28 | 38.53 | 25.75 | 43.12 | 39.17 | 46.53 | 41.57 | 33.31 | 8.48 | - | 3.75 |
| FDP, mg/L  （normal：＜5.0mg/L) | 123.2 | 90.9 | 256.6 | 192.3 | 71.5 | 64.8 | 125.5 | 113.2 | 120.4 | 112.8 | 80.7 | 23.3 | - | 11.3 |
| Total bilirubin, umol/l  （normal:2.0-22.0 umol/l) | 21.5 | 87.2 | 144.9 | 170.1 | 153.9 | 222.7 | 236.1 | 328.6 | 286.5 | 195.5 | 173.4 | 65.7 | - | 49.1 |
| Albumin, g/L  (normal:63.0-82.0g/L) | 13.5 | 23 | 39.2 | 36.1 | 29.9 | 37.4 | 35.8 | 37.5 | 36.2 | 32.5 | 31.4 | 32.6 | - | 31.9 |
| ALT, U/L  (normal:9-52U/L) | 18 | 395 | 1327 | 993 | 775 | 652 | 492 | 449 | 395 | 366 | 334 | 219 | - | 166 |
| AST, U/L  (normal:14-36U/L) | 14 | 695 | 2803 | 2249 | 2255 | 2228 | 1288 | 1321 | 1253 | 1065 | 812 | 286 | - | 179 |
| Carbamide, mmol/l  （normal:2.5-6.1mmol/l) | 4.8 | 10.5 | 14 | 16.6 | 23 | 25.9 | 23.1 | 22.9 | 13.2 | 15.4 | 15.5 | 13.6 | - | 14.7 |
| Creatinine, umol/L  （normal:46-92umol/L) | 101 | 156 | 192 | 160 | 127 | 137 | 97 | 87 | 39 | 45 | 52 | 37 | - | 41 |
| CK, U/L  (normal:30-135U/L) | 918 | 6402 | 8000 | 8000 | 8000 | - | 32000 | 8000 | 30900 | 8000 | 8000 | 3739 | - | 1187 |
| CK-MB, U/L  (normal:0-16U/L) | 17 | 105 | 1500 | 1500 | 1500 | - | 194 | 304 | 350 | 300 | 300 | 75 | - | 55 |
| CK-MM, U/L  (normal:0-137U/L) | 901 | 6297 | 6500 | 6500 | 6809 | - | 31806 | 7696 | 30550 | 7700 | 7700 | 3664 | - | 1132 |
| LDH, U/L  （normal:120-250U/L) | - |  | 2604 | 3105 |  | 3206 | 1864 | 2609 | 1903 | 1573 | 1547 | 957 | - | 882 |
| CTn, ng/ml  （normal:≤0.11 ng/ml） | 0.01 | 0.53 | 1.48 | 0.86 | 0.82 | - | 0.21 | 0.15 | 0.04 | 0.06 | 0.03 | 0.01 | - | 0.02 |
| B type natriuretic peptide precursor, pg/ml  （normal:0-125pg/ml) | 637 | 13000 | 35000 | 35000 | 35000 | - | 35000 | 35000 | 20900 | 14700 | 15900 | 8800 | - | 7600 |
| Myohemoglobin, ng/ml  （normal:7.0-64.0ng/ml) | 2000 | 2000 | 2000 | 2000 | 2000 | - | 2000 | 2000 | 2000 | 2000 | 2000 | 976.6 | - | 555.9 |
| PCT, ng/ml  (normal:＜0.5ng/ml） | - | - | 100 | 100 |  | 64.56 | 39.14 | 25.87 | 10.37 | 8.18 | 5.69 | 2.12 | - | 2.58 |
| WBC, x10^9^/L  (normal:3.5-9.5×10^9^/L) | 2.8 | 10.9 | 34.3 | 19 | 19.4 | 17.6 | 16 | 25.8 | 26.4 | 15.9 | 11.1 | 13.7 | - | 13.4 |
| RBC, x10^12^/L  （normal：3.8-5.1×10^12^/L) | 2.32 | 2.94 | 4.49 | 3.97 | 3.07 | 2.79 | 2.82 | 3.26 | 3.17 | 3.39 | 3.3 | 2.83 | - | 2.79 |
| Hemoglobin, g/L  (normal:115-150g/L) | 49 | 70 | 115 | 79 | 73 | 80 | 73 | 87 | 84 | 88 | 86 | 75 | - | 75 |
| Platelet, ×10^9^/L  (normal:125-350×10^9^/L) | 49 | 47 | 32 | 15 | 13 | 9 | 6 | 65 | 62 | 125 | 83 | 296 | - | 384 |
| CRP, g/L  (normal:0-10.00mg/L) | 10 | 34.8 | 89.32 | 183.13 | 113.92 | 82.8 | 109.82 | 70.61 | 47.47 | 26.86 | 22.16 | 20.77 | - | 39.66 |

INR: international normalized ratio; APTT: activated partial thromboplastin time; FDP: fibrin degradation product; ALT: alanine aminotransferase; AST: aspartate aminotransferase; CK: creatine kinase; CK-MB: creatine kinase-MB;CK-MM: creatine kinase-MM;LDH: lactate dehydrogenase; CTn: cardiac troponin; PCT: procalcitonin; WBC: white blood cell; RBC: red blood cell; CRP: C-reactive protein
