# Supplementary material for: Toxic shock syndrome complicated with symmetrical peripheral gangrene after liposuction and fat transfer: a case report and literature review
Source: BMC Infect Dis. 2021 Nov 6;21:1137. doi: 10.1186/s12879-021-06777-2 (PMC8571909; doi:10.1186/s12879-021-06777-2)
Supplement: Supplementary file 2 — Additional file 2: Centers for Disease Control and Prevention TSS (Other Than Streptococcal) Diagnostic Criteria. [file 12879_2021_6777_MOESM2_ESM.docx]

**Additional file 2. Centers for Disease Control and Prevention TSS (Other Than Streptococcal) Diagnostic Criteria.**

TABLE S2. Centers for Disease Control and Prevention TSS (Other Than Streptococcal) Diagnostic Criteria

| **Clinical Criteria** |
| --- |
| An illness with the following clinical manifestations:   - Fever: temperature greater than or equal to 102.0°F (greater than or equal to 38.9°C) - Rash: diffuse macular erythroderma - Desquamation: 1-2 weeks after onset of rash - Hypotension: systolic blood pressure less than or equal to 90 mm Hg for adults or less than fifth percentile by age for children aged less than 16 years - Multisystem involvement (three or more of the following organ systems): - Gastrointestinal: vomiting or diarrhea at onset of illness - Muscular: severe myalgia or creatine phosphokinase level at least twice the upper limit of normal - Mucous membrane: vaginal, oropharyngeal, or conjunctival hyperemia - Renal: blood urea nitrogen or creatinine at least twice the upper limit of normal for laboratory or urinary sediment with pyuria (greater than or equal to 5 leukocytes per high-power field) in the absence of urinary tract infection - Hepatic: total bilirubin, alanine aminotransferase enzyme, or asparate aminotransferase enzyme levels at least twice the upper limit of normal for laboratory - Hematologic: platelets less than 100,000/mm^3^ - Central nervous system: disorientation or alterations in consciousness without focal neurologic signs when fever and hypotension are absent |
| **Laboratory Criteria for Diagnosis** |
| Negative results on the following tests, if obtained:   - Blood or cerebrospinal fluid cultures (blood culture may be positive for Staphylococcus aureus) - Negative serologies for Rocky Mountain spotted fever, leptospirosis, or measles |
| **Case Classification** |
| - Probable: A case which meets the laboratory criteria and in which four of the five clinical criteria described above are present - Confirmed: A case which meets the laboratory criteria and in which all five of the clinical criteria described above are present, including desquamation, unless the patient dies before desquamation occurs |

TSS: Toxic Shock Syndrome
